# A new and rapid approach for detecting COVID‐19 based on S1 protein fragments

**DOI:** 10.1002/ctm2.90

**Published:** 2020-06-05

**Authors:** Hua Li, Zhe Liu, Yue He, Yingjie Qi, Jie Chen, Yuanyuan Ma, Fujia Liu, Kaisheng Lai, Yong Zhang, Liu Jiang, Xiangdong Wang, Junbo Ge

**Affiliations:** ^1^ Department of Cardiology, Shanghai Institute of Cardiovascular Diseases, Shanghai Xuhui District Central Hospital & Zhongshan‐xuhui Hospital, Zhongshan Hospital Fudan University Shanghai China; ^2^ Department of Medicine BestNovo (Beijing) Medical Technology Co., Ltd Beijing China; ^3^ Department of Cardiology Shanghai Xuhui District Central Hospital & Zhongshan‐xuhui Hospital Shanghai China; ^4^ Division of Life Sciences and Medicine The First Affiliated Hospital of USTC, University of Science and Technology of China Hefei 230000 China; ^5^ Department of Laboratory Medicine Shanghai Xuhui District Central Hospital & Zhongshan‐xuhui Hospital Shanghai China; ^6^ Shanghai Public Health Clinical Center Clinical Trial Institution Shanghai China; ^7^ Institute of Clinical Science, Zhongshan Hospital Fudan University Shanghai China

**Keywords:** biological technology, cardiology

## Abstract

The pandemic of novel coronavirus disease 2019 (COVID‐19) seriously threatened the public health all over the world. A colloidal gold immunochromatography assay for IgM/IgG antibodies against the receptor‐binding domain of severe acute respiratory syndrome coronavirus 2 (SARS‐CoV‐2) S1 protein was established to assess its rapid diagnostic value. We first designed and manufactured all contents of the test cassette of SARS‐CoV‐2 rapid test kit: the colloidal gold‐labeled mouse‐antihuman lgM/lgG antibody, the recombinant SARS‐CoV‐2 antigen, the nitrocellulose membrane control line, and specimen diluents. Furthermore, reverse transcription‐polymerase chain reaction (RT‐PCR) assay, colloidal gold immunochromatography assay, serological validation of cross reaction with other common viruses, and clinical validation were performed. The kit was finally evaluated by 75 serum/plasma samples of SARS‐CoV‐2 infection cases and 139 healthy samples as control, with the result of that the sensitivity, specificity, and accuracy for IgM were 90.67%, 97.84%, and 95.33%, whereas for IgG were 69.33%, 99.28%, and 88.79%, respectively; the combination of IgM and IgG could improve the value: 92.00%, 97.12%, and 95.33%, respectively. Therefore, the rapid detection kit has high sensitivity and specificity, especially for IgM&IgG, showing a critical value in clinical application and epidemic control of COVID‐19.

In December 2019, some unexplained pneumonia cases started to be found in Wuhan, Hubei Province, China. The pathogen was quickly clarified by China researchers as the positive‐sense single‐stranded RNA coronavirus (severe acute respiratory syndrome coronavirus 2 [SARS‐CoV‐2]), belonging to the same family as of severe acute respiratory syndrome coronavirus (SARS‐CoV) and middle east respiratory syndrome coronavirus (MERS‐CoV).^1^ And homology studies showed that SARS‐CoV‐2 had nearly 80% homology with SARS‐CoV and 50% identity with MERS‐CoV, whereas 96.3% identity with a bat's coronavirus.^2^, ^3^ Disease caused by the novel coronavirus was later named as coronavirus disease 2019 (COVID‐19) by the World Health Organization (WHO). COVID‐19 spread rapidly and has brought about a pandemic with more than 4.0 million laboratory confirmed cases until 11 May 2020 (https://covid19.who.int).

COVID‐19 diagnosis requires to be confirmed by SARS‐CoV‐2 nucleic acid detection via RT‐PCR (Reverse Transcription‐Polymerase Chain Reaction) according to WHO COVID‐19 guideline.^4^ However, nucleic acid detection of SARS‐CoV‐2 has obvious limitations in practice.^5^ Further researches indicated that the COVID‐19‐infected patients would also produce specific antibodies by immune response,^6^, ^7^ which was similar to those with SARS‐CoV infection. Based on it, the detection of IgM/IgG in blood became an optional approach to improve the diagnosis, especially for the COVID‐19 patient with negative nucleic acid test result.^8^ For this reason, we designed and developed SARS‐CoV‐2 antibody test reagents. The kit can be performed in the site and took at most 15 minutes to obtain results with only one drop of blood sample, which is more convenient for large population screening and site inspection than nucleic acid test.^9^ Although a large number of antibody detection reagent kits were developed, evidence in terms of the clinical application value was still lacking.^10^ In order to be more beneficial to improve the diagnosis timeliness and accuracy of COVID‐19, we supported following evidence to promote its clinical utility.

**GRAPHICAL HEADLIGHTS**

1. The detection of SARS‐CoV‐2 IgM/IgG takes at most 15 minutes to obtain results with one drop of blood sample.
2. It becomes an optional approach to improve the diagnosis, especially for COVID‐19 patients with negative nucleic acid test.
3. With the development of the database of epidemic investigation for antibody, it might play a valuable role for diagnosis and control of COVID‐19.

We first designed and manufactured all contents of the test cassette of SARS‐CoV‐2 rapid test kit.

1. The contents of the rapid test kit for blood lgM/IgG antibody were designed to include sample soleplate, reaction soleplate, test line (T), control line (C), suction filter paper, and plastic cassette (Figure 1A). Colloidal gold‐labeled mouse‐antihuman IgM/IgG antibody was on the reaction soleplate, the test line was on the NC membrane and covered by recombinant SARS‐CoV‐2 antigen, and the control line, used for quality control, was covered by goat‐antimouse IgM/IgG antibody.
2. Colloidal gold‐labeled mouse‐antihuman lgM/lgG antibody was manufactured by SAIYA Hebei Biotechnology Co., Ltd. To obtain the well‐performance antibody, the antibody was selected for functional test including the positive and negative coincidence rates, minimum test threshold, and accelerated stability. First, the positive and negative coincidence rates were tested. The test line and control line were covered by 1 mg/mL recombinant SARS‐CoV‐2 antigen and 0.5 mg/mL goat‐antimouse lgM/lgG antibody, respectively. Colloidal gold‐labeled mouse‐antihuman lgM/lgG antibody from four different batches were spread on the reaction soleplate for testing by positive reference (P1‐P3) and negative reference (N1‐N6). Colloidal gold‐labeled mouse‐antihuman lgM/lgG antibody evaluated the positive and negative coincidence rates, with samples M1, M3, and M4 showing good coincidence rate in both positive and negative references and selected for further testing (Table S1). Second, minimum test threshold references with dilution of 1:2, 1:6, and 1:18 were used, and M1/M3 fulfilled the requirement of minimum threshold test (Table S2). The test cassettes with M1 and M3 colloidal gold‐labeled mouse‐antihuman lgM/lgG antibody were further tested for the accelerated stability. The test cassettes were kept under dry and closed condition with 37°C temperature for 21 days and tested by using threshold references (Table S3). Finally, the result indicated that M3 fills the requirement.
3. The recombinant SARS‐CoV‐2 antigen was produced according to the procedure.^8^ The recombinant protein was sent to Beijing Jorferin Bio‐Technology Co. Ltd. for further process. To obtain the optimized antigen, strict functional selection steps including the positive and negative coincidence rated, minimum test threshold, and accelerated stability test were carried out. The expressed recombined SARS‐CoV‐2 protein was verified via protein electrophoresis (Figure 1B), demonstrating the same molecular weight as that of predicted size around 30 kDa in the reducing condition. And the reactogenicity of the recombinant antigen was further checked by ELISA using biotin‐labeled anti‐SARS‐CoV‐2 antibodies. It was observed that the recombined SARS‐CoV‐2 antigen bound well with both anti‐SARS‐CoV‐2 antibodies in varied concentration, indicating that the quality of the recombined SARS‐CoV‐2 antigen was viable (Figure 1C). Subsequently, to select the high‐performance recombinant SARS‐CoV‐2 antigen, different batches of recombinant SARS‐CoV‐2 antigen were tested by using positive (P1‐P3) and negative (N1‐N5) references with a concentration of 1 mg/mL (Table S4). Two samples (R1 and R2) out of three obtained 100% positive and negative coincidence rates and were brought to the minimum test threshold inspection with the threshold references in the dilution of 1:2, 1:6, and 1:18. Ultimately, R1 sample obtained 100% positive coincidence rate with 1:2 and 1:6 dilution references and was selected as the final recombinant SARS‐CoV‐2 antigen (Table S5).
4. The nitrocellulose membrane control line was covered by goat‐antimouse lgM/lgG antibody, manufactured by Beijing Jorferin Bio‐Technology Co. Ltd. Colloidal gold‐labeled mouse‐antihuman lgM/lgG antibody covering the control line was also assessed at different concentrations from 5 to 0 mg/mL in a descending order with the same concentration (0.5 μM) of recombinant SARS‐CoV‐2 antigen. We found that as the concentration of the solution gradually decreased, the result turned from positive to negative and finally sample G2 demonstrated better performance than G1 (Table S6).
5. Specimen diluent contained 2.0% trehalose, 2.0% bovine serum albumin, 0.2% ethylenediaminetetraacetic acid disodium, 0.9% sodium chloride, 0.2% proclin 300, and 1.15% 0.5 M pH 7.2 tris buffer.

**Figure 1:**
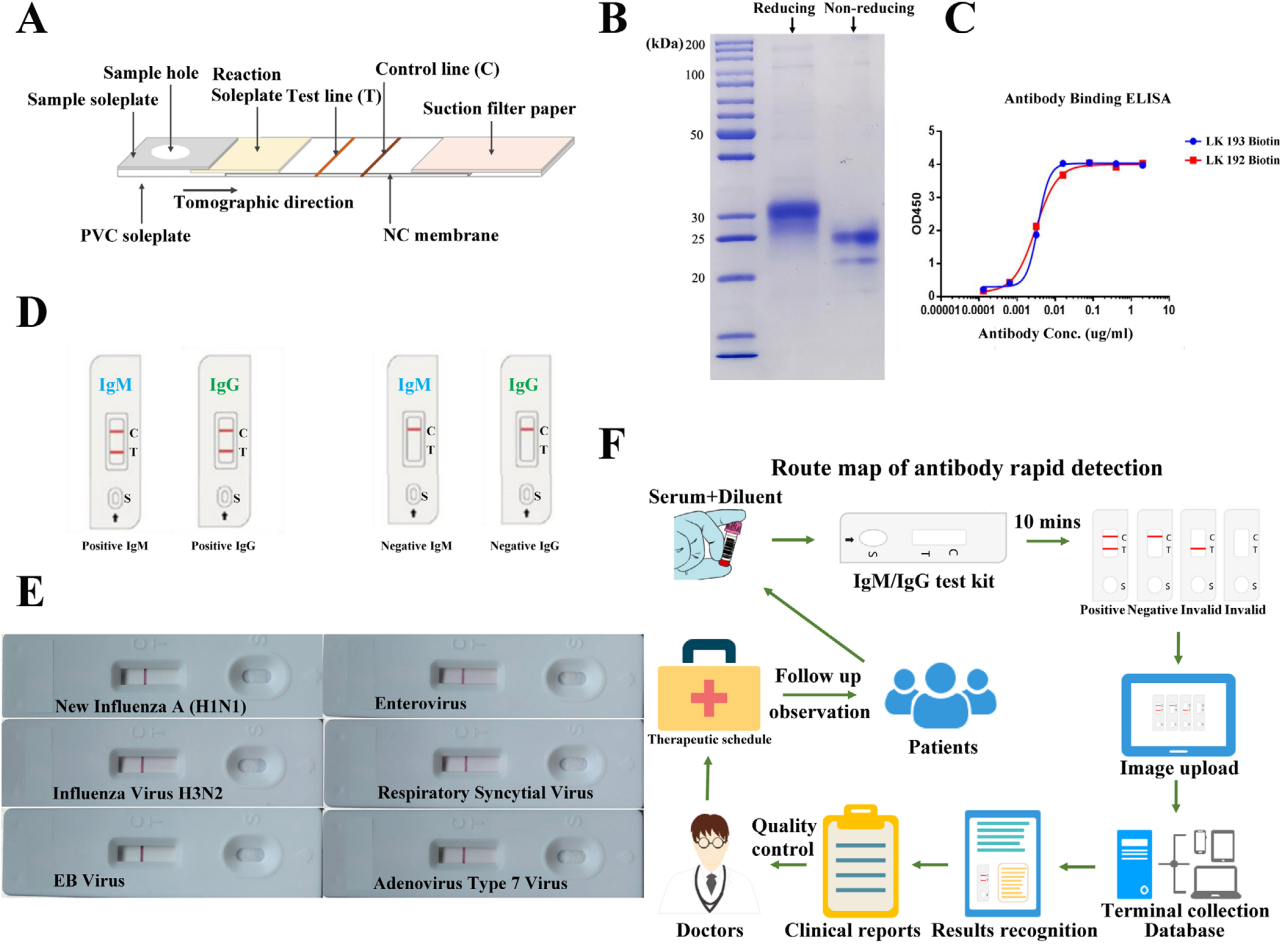
A, SARS‐CoV‐2‐specific IgM/IgG test cassette design. B and C, The protein electrophoresis and ELISA results of recombined SARS‐CoV‐2 antigen. D, The positive and negative results of SARS‐CoV‐2 antibody rapid test kit. E, Cross reaction between SARS‐CoV‐2 and new influenza A (H1N1), influenza virus H3N2, EB virus, enterovirus, respiratory syncytial virus, and adenovirus type 7 virus via SARS‐CoV‐2 IgM antibody test kit. F, The route chart of epidemic investigation for IgM/IgG antibody

Furthermore, RT‐PCR assay,^8^ colloidal gold immunochromatography assay,^8^ serological validation of cross reaction with other common viruses, and clinical validation were performed.

1. Serological validation of cross reaction with other common viruses: The SARS‐CoV‐2 IgM/IgG antibody test was performed by using the kits; the result was determined by the observation of red‐color test line and control line; and the visible red T line and C line indicated positive result, whereas only red C line appearance meant negative (Figure 1D). Serum samples of six patients were selected for the serological cross reaction tests via SARS‐CoV‐2 IgM antibody test kit. The different patient's serum samples including new influenza A (H1N1) virus (2009) (antibody titer 1:10), influenza virus H3N2 (antibody titer 1:12), EB virus (antibody titer 1:10), enterovirus (antibody titer 1:10), respiratory syncytial virus (antibody titer 1:12), and adenovirus Type 7 (antibody titer 1:16) were presented by the Virus Institute of China Center for Disease Control and Prevention (CDC). It was observed that there was no cross reaction between SARS‐CoV‐2 and other common respiratory viruses (Figure 1E).
2. Clinical validation of SARS‐CoV‐2 IgM/IgG rapid test kit: The serum/plasma of 75 COVID‐19 patients in Anhui Provincial Hospital from 31 January to 19 February 2020 and 139 controls from Xuhui Central Hospital were collected for test. All patients (duration from hospitalization to detection: 1‐22 days) were clinically diagnosed as COVID‐19 confirmed with positive SARS‐CoV‐2 nucleic acid test, whereas the controls were healthy with negative nucleic acid test. Healthy group had the median of age 45.5 years, with 18 (12.95%) males, whereas COVID‐19‐comfirmed group had the median age of 47.5 years, with 45 (60%) males; specially, we collected 37 convalescent COVID‐19 cases with median age of 60.5 years and 21 (56.76%) males (Table 1). To prove our kits, the IgM and IgG were tested independently or in combination. At last, the sensitivity, specificity, false positive rate (FPR), false negative rate (FNR), and accuracy were calculated (Table 2): the sensitivity, specificity, and accuracy for IgM were 90.67%, 97.84%, and 95.33%, respectively; whereas for IgG were 69.33%, 99.28%, and 88.79%, respectively. IgM and IgG in combination could improve the value: 92.00%, 97.12%, and 95.33%, respectively. As for 37 convalescent COVID‐19 patients, the nucleic acid and antibody were retested with 35 patients nucleic acid negative and two patients still positive. Among the above 35 patients, four IgM patients already turned to be negative, whereas IgG patients continued to be positive.

**Table 1:** Basal characteristics of COVID‐19 cases and healthy control

	Case group	Controlgroup	Reexamination group
No. of cases	75	139	37
Gender, n (%)			
Male	45 (60.00)	18 (12.95)	21 (56.76)
Female	30 (40.00)	121 (87.05)	16 (43.24)
Age (year), n (%)			
≤16	2 (2.67)	2 (1.44)	1 (2.70)
17‐39	24 (32.00)	102 (73.38)	15 (40.54)
40‐59	34 (45.33)	27 (19.42)	14 (37.84)
≥60	15 (20.00)	8 (5.76)	7 (18.92)
Median (IQR)	47.5 (20.5)	45.5 (15.5)	60.5 (23)
Sample type, n (%)			
Serum	53 (70.67)	139 (100)	26 (70.27)
Plasma	22 (29.33)	0 (0)	11 (29.73)

Abbreviations: IQR, inter quartile range.

**Table 2:** Clinical performance of IgM and IgG tested independently or in combination

	COVID‐19 IgM/IgG antibody rapid test kit					
	IgM		IgG		IgM and IgG	
Real‐time PCR assay	Positive	Negative	Positive	Negative	Positive	Negative
Positive	68	7	52	23	69#	6
Negative	3	136	1	138	4#	135
Subtotal	71	143	53	161	73#	141
Positive percent agreement (sensitivity)	90.67%		69.33%		92.00%	
Negative percent agreement (specificity)	97.84%		99.28%		97.12%	
Positive predictive value (PPV)	95.77%		98.11%		94.52%	
Negative predictive value (NPV)	95.10%		85.71%		95.74%	
False positive rate (FPR)	2.16%		0.72%		2.88%	
False negative rate (FNR)	9.33%		30.67%		8.00%	
Overall agreement (accuracy)	95.33%		88.79%		95.33%	

*Note*. Positive if any of two markers is positive.

In the previous studies, IgM was described as the earliest antibody produced after viral infection, whereas IgG was produced in the recovery phase of viral infection and lasted for several months or years.^11^ Positive IgM antibody usually indicated an acute phase of viral infection, whereas positive IgG antibody suggested late or previous infection. Due to only around 50% positive rate of SARS‐CoV‐2 nucleic acid test^8^, ^12^ under various condition of sample collection and storage, viral infection regions, RNA extraction methods, the quality of nucleic acid detection kit, and so on,^13^ detection of IgM/IgG became a powerful approach for the early diagnosis of COVID‐19 and could help identify the patients with negative nucleic acid but with obvious clinical symptoms.^8^, ^14^ In addition, detection of IgM/IgG can also provide the time course information of viral infection^12^ and predict disease severity in COVID‐19 patients.^15^, ^16^, ^17^ In March 2020, the method of antibody detection gained the official approval in China (China Novel Coronavirus Pneumonia Guideline [Version 7]^18^). Compared with enzyme‐linked immunosorbent assay (ELISA), chemiluminescent immunoassay (CLIA), and colloidal gold immunochromatography,^19^, ^20^ colloidal gold immunochromatography assay could directly test the blood without extraction, which made it well fit for the large‐scale fast test and screening on site.^21^ In the study, IgM and IgG independent test showed high sensitivity (90.67%/ and 9.33%, respectively), high specificity (97.84% and 99.28%, respectively), and high accuracy (95.33% and 88.79%, respectively); especially, combination of IgM and IgG displayed better performance with 92.00% sensitivity, 97.12% specificity, and 95.33% accuracy, which indicated that the products were viable enough for clinical detection of COVID‐19 patients compared with other reagents.^5^ We also reexamined 37 COVID‐19 convalescent patients and found that two patients’ nucleic acid test was still positive with one of them IgM^+^/IgG^+^ and the other IgM^−^/IgG^−^. In the other published studies, the positive nucleic acid test of COVID‐19 convalescent patients was also reported.^22^, ^23^ Therefore, antibody detection could assist to screen COVID‐19‐infected patients accurately, combined with nucleic acid detection.^13^

In sum, the SARS‐CoV‐2 antibody rapid detection kits we demonstrated has high sensitivity and specificity. The reported data suggested that it could be a good prospect for wide application in individual serological qualitative monitoring and might play a valuable role in practical applications for the diagnosis and epidemic control of COVID‐19, with the development of the big database of epidemic investigation for SARS‐CoV‐2 IgM/IgG antibody (Figure 1F).

## CONFLICT OF INTEREST

The authors declare no conflict of interest.

## Supporting information

Supporting information
