# Supplementary material for: A new and rapid approach for detecting COVID‐19 based on S1 protein fragments
Source: Clin Transl Med. 2020 Jun 5;10(2):e90. doi: 10.1002/ctm2.90 (PMC7427819; doi:10.1002/ctm2.90)
Supplement: Supplementary file 1 — Supporting information [file CTM2-10-e90-s001.docx]

**Supplementary Tables**

**Table 1. Positive and negative coincidence rate of colloidal gold-labeled mouse-anti-human lgM/lgG antibody**

| **Samples** | **References** | | | | | | | | |
| --- | --- | --- | --- | --- | --- | --- | --- | --- | --- |
|  | **P1** | **P2** | **P3** | **N1** | **N2** | **N3** | **N4** | **N5** | **N6** |
| M1 | + | + | + | - | - | - | - | - | - |
| M2 | + | - | + | - | - | - | - | - | - |
| M3 | + | + | + | - | - | - | - | - | - |
| M4 | + | + | + | - | - | - | - | - | - |

Positive, +; Negative, -

**Table 2. Minimum test threshold Colloidal gold-labeled mouse-anti-human lgM/lgG antibody minimum test threshold result**

| **Samples** | **Dilution** | | | | | | | | |
| --- | --- | --- | --- | --- | --- | --- | --- | --- | --- |
|  | **1:2** | | | **1:6** | | | **1:18** | | |
| M1 | + | + | + | + | + | + | - | - | - |
| M3 | + | + | + | + | + | + | - | - | - |
| M4 | + | + | + | + | + | - | - | - | - |

Positive, +; Negative, -

**Table 3. Accelerated stability test result of colloidal gold-labeled mouse-anti-human lgM/lgG antibody**

| **Samples** | **Dilution** | | | | | | | | |
| --- | --- | --- | --- | --- | --- | --- | --- | --- | --- |
|  | **1:2** | | | **1:6** | | | **1:18** | | |
| M1 | + | + | + | + | － | + | - | - | - |
| M3 | + | + | + | + | + | + | - | - | - |

Positive, +; negative, -.

**Table 4. Positive and negative coincidence rate test of recombinant SARS-CoV-2 antigen**

| **Sample** | **References** | | | | | | | |
| --- | --- | --- | --- | --- | --- | --- | --- | --- |
|  | **P1** | **P2** | **P3** | **N1** | **N2** | **N3** | **N4** | **N5**  **-**  **-**  **-** |
| R1 | + | + | + | - | - | - | - | - |
| R2 | + | + | + | - | - | - | - | - |
| R3 | + | - | + | - | - | - | - | - |

Positive, +; negative, -.

**Table 5. Minimum test threshold of recombinant SARS-CoV-2 antigen**

| **Sample** | **Dilution** | | | | | | | | |
| --- | --- | --- | --- | --- | --- | --- | --- | --- | --- |
|  | **1:2** | | | **1:6** | | | **1:18** | | |
| S1 | + | + | + | + | + | + | - | - | - |
| S2 | + | + | + | － | + | + | - | - | - |

Positive, +; negative, -.

**Table 6. The reaction activity result of goat-anti-mouse lgM/lgG antibody and recombinant SARS-CoV-2 antigen**

| **IgM/IgG concentration（mg/mL）** | **Result** | |
| --- | --- | --- |
|  | **G1(6.8mg/ml)** | **G2(6.6mg/ml)** |
| 5.0 | + | + |
| 2.5 | + | + |
| 1.25 | + | + |
| 0.64 | + | + |
| 0.32 | - | + |
| 0.16 | - | - |
| 0.08 | - | - |
| 0.04 | - | - |
| 0.02 | - | - |
| 0.01 | - | - |
| 0.005 | - | - |
| 0 | - | - |

Positive, +; negative, -.
